# Identification of four latent classes of acute respiratory distress syndrome using PaO_2_/F_I_O_2_ ratio: an observational cohort study

**DOI:** 10.1038/s41598-024-52243-9

**Published:** 2024-01-23

**Authors:** Calvin Loewen, Brenden Dufault, Owen Mooney, Kendiss Olafson, Duane J. Funk

**Affiliations:** 1 Department of Anesthesiology, Perioperative and Pain Medicine, University of Manitoba, Winnipeg, MB Canada; 2 Department of Community Health Sciences, University of Manitoba, Winnipeg, MB Canada; 3 George and Fay Yee Centre for Healthcare Innovation, University of Manitoba, Winnipeg, MB Canada; 4 Department of Internal Medicine, Section of Critical Care, University of Manitoba, Winnipeg, MB Canada; 5 Departments of Anesthesiology and Medicine, Section of Critical Care, Max Rady College of Medicine, University of Manitoba, 2nd Floor Harry Medovy House, 671 William Avenue, Winnipeg, MB R3E 0Z2 Canada

**Keywords:** Physiology, Respiration

## Abstract

Biological phenotypes in patients with the acute respiratory distress syndrome (ARDS) have previously been described. We hypothesized that the trajectory of PaO_2_/F_I_O_2_ ratio could be used to identify phenotypes of ARDS. We used a retrospective cohort analysis of an ARDS database to identify latent classes in the trajectory of PaO_2_/F_I_O_2_ ratio over time. We included all adult patients admitted to an intensive care unit who met the Berlin criteria for ARDS over a 4-year period in tertiary adult intensive care units in Manitoba, Canada. Baseline demographics were collected along with the daily PaO_2_/F_I_O_2_ ratio collected on admission and on days 1–7, 14 and 28. We used joint growth mixture modeling to test whether ARDS patients exhibit distinct phenotypes with respect to both longitudinal PaO_2_/F_I_O_2_ ratio and survival. The resulting latent classes were compared on several demographic variables. In our study group of 209 patients, we found that four latent trajectory classes of PaO_2_/F_I_O_2_ ratio was optimal. These four classes differed in their baseline PaO_2_/F_I_O_2_ ratio and their trajectory of improvement during the 28 days of the study. Despite similar baseline characteristics the hazard for death for the classes differed over time. This difference was largely driven by withdrawal of life sustaining therapy in one of the classes. Latent classes were identified in the trajectory of the PaO_2_/F_I_O_2_ ratio over time, suggesting the presence of different ARDS phenotypes. Future studies should confirm the existence of this finding and determine the cause of mortality differences between classes.

## Introduction

The mortality of ARDS remains between 37 and 48%^1,2^. The disappointing results of many clinical trials is possibly due to different ARDS phenotypes resulting from the heterogeneous causes of ARDS. Identifying these different ARDS groups early may result in an improved treatment effect within randomized trials^3,4^.

Several studies have attempted to identify ARDS phenotypes by using biological markers^5–7^. These studies have identified several ARDS phenotypes and, in reanalyzing some of the previous interventional ARDS trials, found outcome differences based on the underlying phenotype. In the Fluid and Catheter Treatment Trial, the intention to treat trial found no difference in survival^8^. However, when data from this trial were re-analyzed comparing patient groups based on a two class sub-phenotype model of ARDS, a mortality difference was present^9^. Similarly, in a trial comparing simvastatin with placebo in ARDS patients, the initial trial was negative for a mortality difference, but with re-analysis of the data utilizing the two-phenotype model, the hyperinflammatory phenotype showed a reduced mortality with simvastatin administration^10,11^.

These recent studies of ARDS phenotypes have utilized a statistical technique called latent class analysis (LCA). LCA is a type of mixture modeling used to find hidden clusters among multivariate data, based on the hypothesis that the observed variance and patterns are caused by several unobserved groups or classes. We hypothesized that longitudinal lung function data may also demonstrate latent groupings (trajectories) in patients with ARDS.

Our hypothesis was that there are phenotypes that can be discovered using PaO_2_/F_I_O_2_ ratio over time in patients with ARDS. We conducted a retrospective cohort analysis of our institutional ARDS database to determine, as our primary outcome, if there were latent classes present in the trajectories of PaO_2_/F_I_O_2_ ratio. Secondary outcomes were to determine what, if any, differences were present between the different classes that were discovered.

## Results

Two hundred and nine (209) patients met inclusion criteria and had complete data for analysis. Baseline demographics of the patients are presented in Table 1 and are grouped as those who survived and those who died. There were 121 survivors and 88 patients who died for a mortality rate of 42.1%.

**Table 1:** Baseline demographics in the cohort of ARDS patients, by those who died and who survived.

	Alive (121)	Dead (88)	p value
Age (years)	46 [36–61]	60 [49–71]	< 0.01
APACHE II score	21 [16–26]	27 [22–34]	< 0.01
Charlson co-morbidity score	2 [1–3]	2 [2–4]	0.267
PaO_2_: F_I_O_2_ ratio day 1	114 [83–158]	119 [73–170]	0.56
Compliance (day 1) (ml/cmH_2_O)	32 [25–38]	26 [22–32]	0.01
Vt/Kg day 1 (ml)	5.8 [4.6–7.7]	6.2 [5.3–7.9]	0.37
PEEP day 1 (cmH_2_O)	10 [8–14]	10 [8–12]	0.04
Plateau pressure day 1 (cmH_2_O)	28 [23–30]	30 [26–32]	0.13
Days ventilated	11 [6–17]	7 [2–14]	< 0.01
ARDS etiology (%)			
Pneumonia	81%	66.3%	0.01
Non-lung sepsis	9.5%	15.6%	0.20
Other	9.5%	18.1%	0.11

Days ventilated refers to mechanical ventilation only.

*APACHE* acute physiology, age and chronic health evaluation score, *PEEP* positive end expiratory pressure.

Patients who died were older and had a higher APACHE II score than those who survived, although the Charlson co-morbidity score was similar between patient classes. PaO_2_/F_I_O_2_ ratio and pulmonary compliance on day 1 were slightly lower in the group who died. Ventilatory parameters (tidal volume/kg ideal body weight, PEEP, and plateau pressure) were similar between groups, and consistent with current ARDS ventilator management guidelines. The dominant etiology of ARDS was pneumonia, with non-lung sepsis comprising the next largest etiological category.

The Bayesian Information Criteria (BIC) suggested that a 4-class model provided the best fit. Table 2 shows the BIC and the posterior classification probabilities for each class. The 4-class model had the lowest BIC with a 5-class model demonstrating a slightly higher BIC.

**Table 2:** Bayesian Information Criterion (BIC) and percentage of patients delegated to that class.

Number of classes	BIC	% Class 1	% Class 2	%Class 3	%Class 4	% Class 5
1	14717	100.00				
2	14555	79.90	20.10			
3	14509	11.48	57.42	31.10		
4	14490	11.96	24.40	46.89	16.75	
5	14500	22.01	16.27	20.10	10.05	31.58

The optimal BIC predicted a four-class model.

The posterior classification probability is a key indicator of model fit and is shown in Table 3. This is each subjects’ estimated probability of belonging to each latent class, based on their unique observations of PaO_2_/F_I_O_2_ ratio and survival. If the model has extracted well-separated and predictive latent classes, each subject should map with high probability to one latent class only and have low probability elsewhere. As can be seen in Table 3, the classes seem well separated, with average posterior classification probabilities for all classes exceeding 0.8.

**Table 3:** Posterior classification probabilities.

Probability of each class				
Class number	1	2	3	4
1	0.9300	0.0151	0.0011	0.0538
2	0.0132	0.8586	0.0652	0.0630
3	0.0087	0.0430	0.9256	0.0228
4	0.0591	0.0648	0.0729	0.8032

Numbers represent each subject’s estimated probability of belonging to each latent class, based on their unique set of observations. If the model has extracted well-separated and predictive latent classes, each subject should map with high probability to one latent class only and have low probability elsewhere.

**Figure 1:**
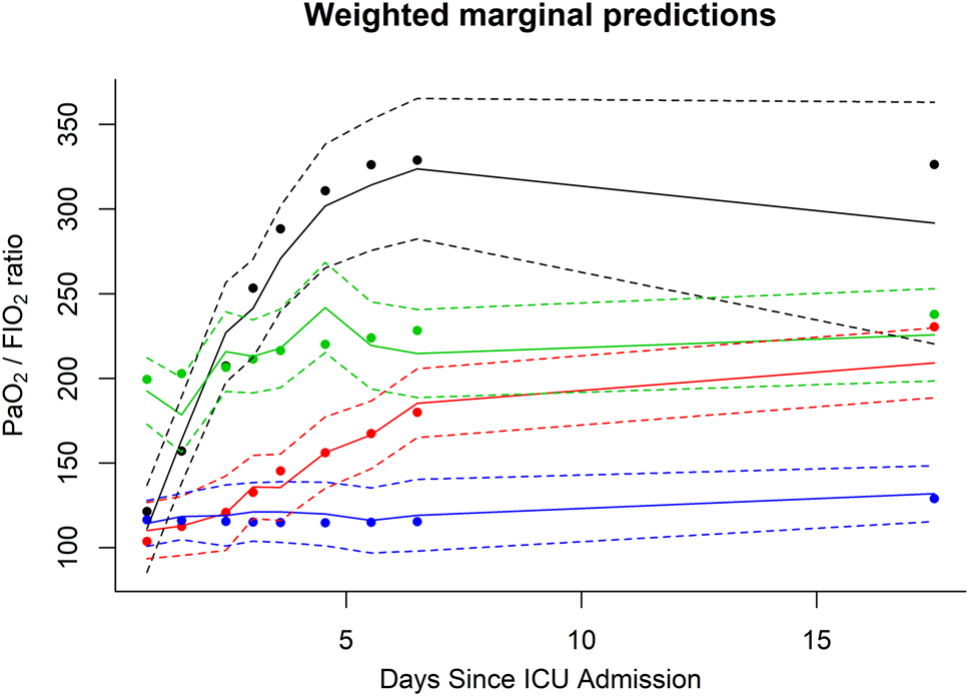
Estimated latent trajectories for the 4 different classes based on PaO_2_/F_I_O_2_ ratio. Values are plotted with their 95% confidence interval. Class 1: black; class 2: red; class 3: green; class 4: blue.

Figure 1 shows the PaO_2_/F_I_O_2_ ratio trajectory for the 4 latent classes. All the classes have an initial PaO_2_/F_I_O_2_ ratio consistent with moderate to severe ARDS. Two of these three classes (class 1, black and 2, red) have an increase in their PaO_2_/F_I_O_2_ ratio over time, with one (black) having a significant increase in PaO_2_/F_I_O_2_ ratio within the first 3 days. One class has a PaO_2_/F_I_O_2_ ratio that fails to improve during the hospital stay (class 4, blue). The final class (class 3, green) began with a PaO_2_/F_I_O_2_ ratio that would be defined as moderate (borderline mild) ARDS and fails to show an improvement in their PaO_2_/F_I_O_2_ ratio over time.

**Figure 2:**
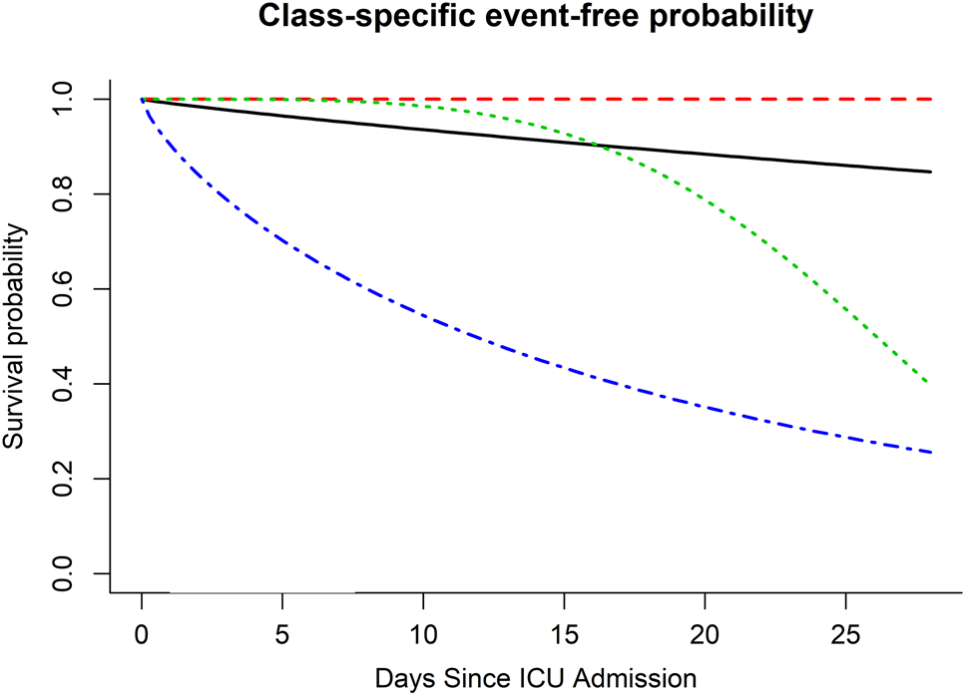
Class specific event free survival probability for the different classes. Class 4 (blue), the class with the second lowest PaO_2_/F_I_O_2_ ratio, the highest APACHE score, and which showed no improvement over time had the lowest survival probability of all 4 classes. The two classes that showed significant improvement in their PaO_2_/F_I_O_2_ ratio within the early phase of their ARDS (class 1 black and class 2, red) showed the best survival. Class 3 (green) had the least severe form of ARDS based on initial PaO_2_/F_I_O_2_ ratio and did not show an improvement in their PaO_2_/F_I_O_2_ ratio and showed a survival trend that mimicked the patient class with the most severe form of ARDS (class 4 blue). The hazard function for this class increased exponentially past day 10.

The survival probability of the different classes is shown in Fig. 2. Class 4 (blue), the class with the second lowest PaO_2_/F_I_O_2_ ratio, the highest APACHE score, and which showed no improvement over time had the lowest survival probability of all 4 classes. The hazard ratio for this class was highest within the first 5 days and then began to level off. The two classes that showed significant improvement in their PaO_2_/F_I_O_2_ ratio within the early phase of their ARDS (class 1 black and class 2, red) showed the best survival, with their hazard ratio for death showing low initial rates that continued throughout the study period.

The final class (class 3 green) was the class that began with what appeared to be the least severe form of ARDS based on initial PaO_2_/F_I_O_2_ ratio. This class did not show an improvement in their PaO_2_/F_I_O_2_ ratio and showed a survival trend that mimicked the patient class with the most severe form of ARDS (class 4 blue), based on PaO_2_/F_I_O_2_ ratio. The hazard function for this class increased exponentially past day 10.

To determine if there were any clinical differences between the groups, we analyzed patient demographics between the 4 latent classes. The goal was to determine how the classes differed with the hope of explaining and predicting class membership.

We first calculated each subject’s posterior classification probability, which tells us their chance of belonging to each latent class, given their longitudinal and survival data. We then assigned each subject to their likeliest class, and treated it as observed. This process is known as modal assignment. Since the posterior probabilities are high for each likeliest class, this imparts tolerable amounts of error for this process. The results of this analysis including key variables is presented in Table 4.

**Table 4:** Comparison of baseline demographic and physiological parameters between the 4 latent classes.

	Class				
	1	2	3	4	Test
Number (%)	25 (12.0%)	50 (23.9%)	34 (16.3%)	100 (47.8%)	
APACHE II score	23 [16–31]	22 [18–26]	20 [15–26]	25 [20–32]	0.008
Charlson_Score	3 [2–5]	2 [1–3]	3 [2–4]	2 [1–4]	0.076
P/F ratio day 1	111 [66–138]	99 [70–139]	185 [173–228]	102 [72–143]	< 0.001
PEEP day 1	10 [10–12]	11 [10–14]	8 [5–10]	10 [8–12]	0.003
P_Plat_ day 1	29 [23–32]	28 [26–30]	27 [23–30]	29 [26–31]	0.556
V_t_/kg day 1	6 [5, 6]	5 [4–7]	8 [6–9]	6 [5–7]	< 0.001
Compliance day 1	23 [16–35]	31 [26–38]	26 [25–32]	26 [23–34]	0.243
ICU LOS	8 [6–13]	15 [11–24]	15 [7–20]	12 [3–20]	< 0.001
Days intubated	5 [3–10]	12 [9–16]	10 [5–15]	10 [3–17]	0.003
Age (years)	46 [28–55]	48 [42–60]	59 [41–72]	56 [39–67]	0.035
Withdrawal of care	2 (8.0%)	2 (4.0%)	10 (29.4%)	50 (50.0%)	< 0.001
28 day mortality	1 (0.5%)	2 (1%)	9(4.3%)	42 (20%)	< 0.001
Female	14 (56.0%)	30 (60.0%)	14 (41.2%)	45 (45.0%)	0.224

*APACHE II score* acute physiology, age and chronic health evaluation II, *P/F ratio* PaO_2_/F_I_O_2_ ratio, *PEEP* positive end expiratory pressure (cmH_2_O), *P*_*plat*_ plateau pressure (cmH_2_O), *Vt/kg* tidal volume (cc)/kilogram ideal body weight (kg), *ICU LOS* intensive care unit length of stay.

**Figure 3:**
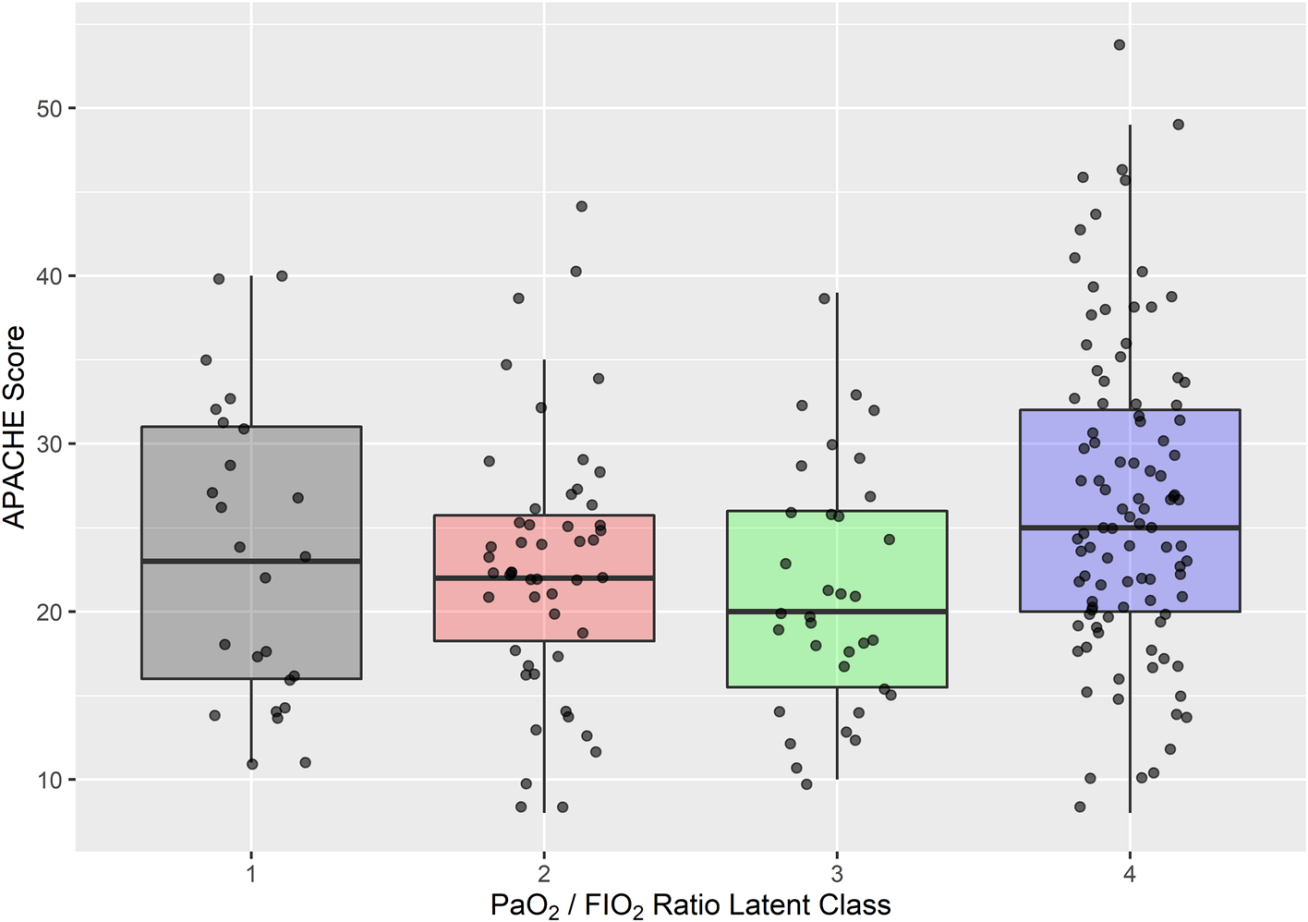
Acute physiology, age and chronic health evaluation II (APACHE) score between classes. Class 3 (green) had the lowest score of all classes, suggesting reduced severity of disease. p = 0.008 Kruskal–Wallis rank sum test. This is despite this class having a decreased survival probability when compared with class 1 and class 2.

The most interesting class to examine in this regard is class 3 (green). This is the class that initially had a PaO_2_/F_I_O_2_ that would be categorized as moderate (borderline mild) ARDS and was higher than all the other classes yet failed to improve during their ICU stay. This class had the lowest APACHE II score of all 4 classes (Fig. 3), and the distribution of this variable differed significantly between latent classes (Kruskal–Wallis chi-squared p = 0.008). This suggests a greater severity of non-pulmonary disease than the other classes. This class was also older than the other classes (data not shown; Kruskal–Wallis chi-squared p = 0.02).

**Figure 4:**
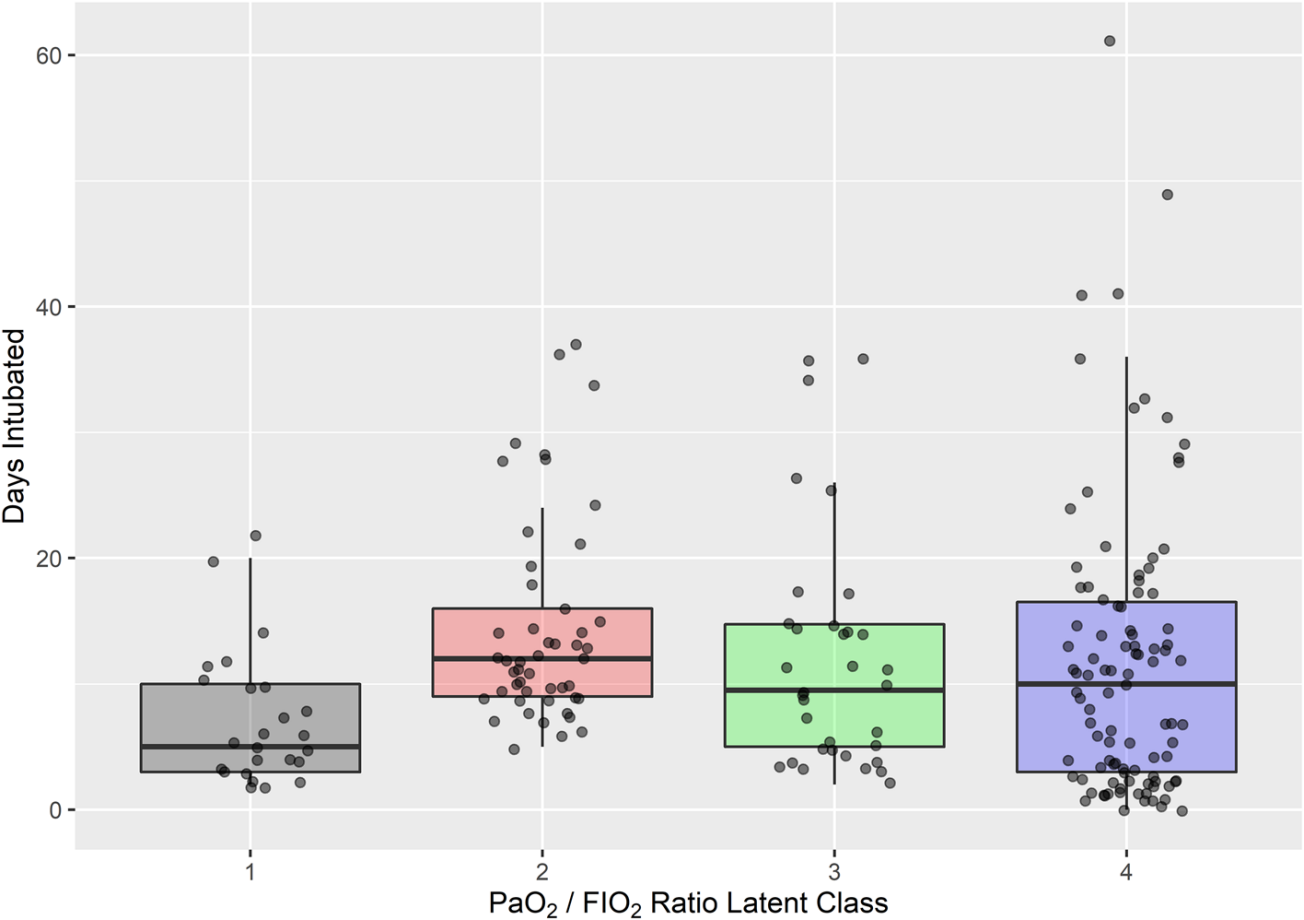
Difference in days intubated between classes. There was a significant difference between classes (p = 0.003, Kruskal–Wallis rank sum test), with class 1 having the shortest intubation time. When class 1 was removed from analysis, the difference became non-significant between classes (data not shown).

The number of days intubated did differ between classes, with class 1 (black) showing the shortest length of intubation amongst the classes (Fig. 4, Table 4, Kruskal–Wallis p = 0.003). However, this difference in days intubated disappeared if class 1 was excluded. This suggests that class 1 had a milder form of ARDS that improved rapidly. This is confirmed by the PaO_2_/F_I_O_2_ trajectory and hazard ratio in Figs. 1 and 3.

When examining other demographic variables, there was no difference between the classes with respect to distribution of sex (Fisher’s exact test p value = 0.2). There was a significant difference between classes, however, with respect to the rate at which care was withdrawn. Patients in class 4 (blue) experienced withdrawal of life-sustaining therapies at a significantly higher rate than those in the other latent classes (Fisher’s exact test p < 0.001). When comparing class 4 (blue) with class 2 (red, the class with the lowest baseline PaO_2_/F_I_O_2_ ratio) the unadjusted odds ratio of having care discontinued was 11.2 times higher in class 4 than in class 2 (95% CI 2.7–52.5, p < 0.001). The reason for what precipitated the decision to withdraw life sustaining therapies in patients in this study was not available to us. However, when using multivariable logistic regression using latent class, day 1 P_a_O_2_/F_I_O_2_ ratio, day 1 compliance, APACHE II score, age and Charlson co-morbidity score as predictor variables we found that the odds ratio of withdrawal of care were not different between the 4 groups, utilizing the black class as the baseline. In fact, none of the predictor were significantly associated with withdrawal of care.

## Discussion

Our study demonstrated that when assessing the trend of PaO_2_/F_I_O_2_ ratio over time, 4 latent classes of ARDS were present. The 4-class model proved the most robust based on the BIC (Table 2). These 4 classes had differing trajectories in their PaO_2_/F_I_O_2_ ratio (Fig. 1). Class 1 (black) had a rapid increase in their PaO_2_/F_I_O_2_ ratio over the first 5 days of mechanical ventilation. Class 2 (red) had a slow, but demonstrable increase in their PaO_2_/F_I_O_2_ ratio, but this increase was not as high as class 1 (black). Class 3 (green) started off with the mildest form of ARDS based on PaO_2_/F_I_O_2_ ratio but failed to show an increase for the duration of the study. Class 4 (blue) failed to improve their PaO_2_/F_I_O_2_ ratio throughout the observation period.

The prevalence of each class was different with the rapid improvement class (class 1, black) having the lowest frequency of at 12%. The most common class (class 4, blue) comprised 47% of the patient population.

The survival probability between these 4 classes were also different (Fig. 2). For classes 1 (black) and 2 (red, early improvement in PaO_2_/F_I_O_2_ ratio), the hazard ratio for death was low throughout the observation period. This contrasts with class 4 (blue) who had a high risk of death early in their ARDS course, presumably due to early worsening of their condition, with a subsequent decrease and then plateau in their hazard ratio for death. The most interesting class (class 3, green) had an exponential increase in their hazard of death beginning at day 10. This is interesting as this class had the highest PaO_2_/F_I_O_2_ ratio at baseline yet failed to show an improvement in this ratio throughout the duration of the observation period.

The patients in class 3 (green) comprised the second fewest number of patients in our study (16%). These patients were older than patients in the other classes but had the lowest mean APACHE score. The patients in class 4 (blue) did not have a significant difference in the number of days intubated when compared to the other classes (in exclusion of class 1, black) but had withdrawal of life sustaining therapy at a significantly higher rate than those patients in the other classes. However, when multivariable logistic regression was performed, there was no difference between groups in the odds ratio of withdrawal of care when correcting for latent class, day 1 P_a_O_2_/F_I_O_2_ ratio, day 1 compliance, APACHE II score, age and Charlson co-morbidity score.

Clinicians caring for patients with ARDS have long recognized that ARDS is not a homogenous disease^12,13^. Over the past 5 years there have been numerous studies that have utilized latent class analysis to determine if there are differing ARDS phenotypes^5–7,10^. These studies have primarily used biomarkers and baseline physiologic and demographic parameters to delineate their latent classes. These studies, in aggregate, have demonstrated either 2 or 3 different phenotypes, and can be broadly categorized as either hyperinflammatory/hypotensive or lower inflammatory/hemodynamically stable phenotypes. In these studies, which were secondary analysis of earlier ARDS trials, a differential response to therapeutic interventions (notably fluid administration, simvastatin use and PEEP) were found, even though in the initial trials the intervention was not shown to be beneficial^7,9,10,14^. These findings have led to the hope that ‘personalized medicine’ for ARDS may be forthcoming^3,4,15^.

Our study differed from those done previously in that it utilized longitudinal data with a readily available bedside measurement (PaO_2_/F_I_O_2_ ratio). This allowed us to leverage the full information in our data and focus on the association between the longitudinal lung function measures and survival, which is treated not as binary but true time-to-event. Previous studies have shown that the baseline PaO_2_/F_I_O_2_ ratio has not been useful in discriminating between the different ARDS phenotypes^6^. Our longitudinal data of this parameter, however, does delineate 4 different classes of ARDS, suggesting the importance of the PaO_2_/F_I_O_2_ ratio trend versus baseline values. Our results demonstrate a more real-world experience of the clinical trajectory of ARDS patients, as compared to the previous studies that utilized data from clinical trials. The ventilatory management of ARDS at our institutions was in keeping with the latest consensus strategies for the management of ARDS^16^.

Previous work utilizing plasma biomarkers and latent class analysis to determine different ARDS phenotypes has the goal of personalizing the management of ARDS with respect to fluid, PEEP levels and other therapeutic interventions. The longitudinal PaO_2_/F_I_O_2_ ratio data from our study and its relation to mortality does not have a specific therapeutic intervention option, but it does show that there are latent classes in the trajectory of ARDS patients. This information is biologically interesting in that it shows that the trajectory of ARDS, with respect to the PaO_2_/F_I_O_2_ ratio was not significantly different between the 4 classes after approximately the 5th day after admission. This suggests that any clinical intervention for this disease should be administered early in the course to have maximum therapeutic benefit.

Limitations to our study, including a smaller sample size than previous latent class ARDS work, are those expected with any retrospective study. These include the retrospective nature of the data collection, missing data, reason for withdrawal of life sustaining therapy and classification bias of ARDS. Previous work has not identified latent classes of ARDS classes based on PF ratios. This ratio can be altered by many factors that may not be related to underlying biological phenotypes. Further work with a larger sample size needs to be conducted to ensure the veracity of our findings. The strengths of our study are that is a more ‘real world’ scenario for ARDS phenotypes, which differentiates it from previous work that was a secondary analysis from carefully controlled randomized trials.

## Methods

Research ethics board approval from The University of Manitoba Biomedical Research Ethics Board was obtained (H2017:129(HS20716)). All methods were carried out in accordance with relevant institutional guidelines and regulations. As all the data were collected anonymously and deidentified, the research ethics board (The University of Manitoba Biomedical Research Ethics Board, reference number: H2017:129(HS20716)) waived written informed consent from participants. Our primary data sources were the Winnipeg Regional Health Authority (WRHA) Critical Care Database and the WRHA ARDS audit database, an ongoing audit project that contains information on adult ICU patients in our health region who were given a diagnosis of ARDS, using the Berlin criteria^17^. This database provided the longitudinal measures of gas exchange and respiratory mechanics data.

The PaO2/F_I_O_2_, (PF) ratio was calculated using daily PaO_2_ and F_I_O_2_ from the morning arterial blood gas. We chose this time period to sample arterial blood to calculate the PF ratio for consistency. As part of institution protocol blood gases are ordered daily for the first 7 days of a mechanically ventilated patients ICU stay, and blood gas ordering after this point is at the discretion of the attending clinician. These data were collected daily on admission days 1–7, 14 and 28. The database was audited by an independent observer to ensure all patients met the Berlin criteria for ARDS. We examined survival to 28 days, with all patients beyond that time treated as censored with respect to survival.

### Ethics approval

Research ethics board approval from The University of Manitoba Biomedical Research Ethics Board was obtained (H2017:129(HS20716)). All authors contributed equally to this work and meet the requirements for authorship.

## Statistical methods

Longitudinal data can be clustered into potential phenotypes using growth mixture models (GMMs), which assume that growth characteristics such as the intercept and rates of change differ between a fixed number of unobserved subpopulations^18^. Subjects are allowed to vary randomly around their cluster-specific trajectory according to conventional linear mixed-effects models, which are estimated alongside the latent clustering task^19^. Thus, subject-specific information is preserved in addition to obtaining insights about phenotypic trajectories.

Joint latent class mixed models (JLCMM), in which the growth mixture and survival processes are estimated simultaneously, offer several important advantages. First, it mitigates missing data bias^20^. Secondly, joint models are more statistically efficient than other approaches (e.g. Cox survival models) with the longitudinal process used as a time-varying covariate^21^. Most advantageously, the JLCMM provides unique hazard functions and survival curves for each of the latent trajectories in a single framework that does not ignore the uncertainty in latent class membership.

JLCMMs were estimated using version 1.7.9 of the R package ‘lcmm’^22^. Each latent trajectory was fit with a quadratic effect for time (days in ICU, described above) and random intercepts for all subjects. Models with a cubic effect for time were also explored but provided similar inferences and are not reported. The class-specific hazards were modeled using the Weibull distribution, which allows the risk of death to vary non-linearly over time. To determine the number of classes, we created models with one to five latent classes and compared them using the Bayesian Information Criterion, as well as clinical plausibility and estimated class size; small classes may be unreliable and a sign of overfitting. Each of the five competing models was run 15 times with random parameter initializations using the ‘grid search’ function to avoid local minima of the log-likelihood^23^. Residual distributions were examined with scatterplots and quantile–quantile plots.

The latent classes were compared with respect to APACHE II score, number of days intubated, sex, and risk of withdrawal of life sustaining therapy. Univariate associations between these factors and the latent trajectories were tested by adding them as covariates to the JLCMM. We report p-values from these tests, which account for class membership uncertainty. We also assigned subjects to their most likely class and plotted them against each covariate^18^.

As we did not know how many classes may be present, and the nature of LCA, a sample size was not calculated. All patients were analyzed on a complete case basis and those cases with missing data were excluded. Data are presented as mean ± standard deviation for normally distributed data and as median [Interquartile range] for non-normally distributed data. p values are reported as two tailed. Between group comparisons were performed using a Students t test or Mann–Whitney test. Categorical data was analyzed using Fisher’s exact test. Normality was assessed using the Kolmorgorov–Smirnov test, and logistic regression was performed with robust standard errors. P values less than 0.05 were considered significant.

## Data Availability

The datasets generated and/or analyzed during the current study are not publicly available due to our local biomedical research ethics board restrictions, but are available from the corresponding author on reasonable request.
